# The Hospital Problem

**Published:** 1886-10-16

**Authors:** 


### THE HOSPITAL PROBLEM.

By The Right Hon. the EARL OF DERBY.

The Utility and Necessity of Hospitals.

I have been asked to address to you a few observations, and though I am not very fond of adding to that torrent of speech, to that cataract of chatter, which is poured upon us every day and upon every possible subject, the more especially as the subject is one on which it is impossible to say anything which is not familiar to those who read their newspapers, still, under all the circumstances, I think it would be uncourteous in me to refuse. I suppose we are all agreed as to the utility and necessity of hospitals. They are institutions, as thousands of persons have said before, by which the richer classes, who never enter them as patients, profit as much as the poor who do; for they are schools of medical science, which without them could be studied only at the utmost disadvantage. It is needless to speak in defence of hospitals, but it is no doubt a question, which is open to discussion, whether special hospitals limited to one particular class of patients are desirable. I think myself that the multiplication of them tends to become excessive, and that where they are set up on a very small scale they cause a certain waste of funds. But I do not see that that applies to the case of hospitals established for the relief or mitigation of a disease so general as consumption. I have been for many years president of the Hospital for Consumption at Brompton, and great as have been the sums expended on that institution, I doubt whether by any other investment of capital and energy it would have been possible to do more for the relief of human suffering. Assuming hospitals to be necessary, how ought they to be maintained? There are only four ways. We may depend on endowments, on subscriptions, on payments by patients, or by public aid in the shape of grants either from local rates or general taxation. No one would meddle with the endowments which already existed, but new ones cannot speedily be established on a large scale. Assistance from rates is not in principle objectionable, for a hospital has certainly as large a claim, on the score of public utility, as a school of drawing or of music; but that is a resource which is heavily drawn upon already for many purposes, and it does not seem desirable to increase the public burdens. The problem, therefore, was how to make charity and self-help not antagonistic, as on the face of matters they were apt to appear, but supplemental one to the other.

The Facts Stated.

Nobody doubts that a great many people get gratuitous medical help, who ought, in part at least, to provide it for themselves. Nobody, on the other hand, can deny that among the working classes there are immense numbers of persons who, with the best will to do so, cannot meet the requirements of illness in the family in ordinary times. They can just make the two ends meet, and no more. Sometimes they cannot even do that. We are, in short, face to face with the old eternal difficulty—how to help without pauperising, how to be kind without being unjust, and just without being unkind. For my part, I do not pretend to be able to solve the difficulty. Human life is too complex to be regulated by any single and simple formula. We can only deal with it in a tentative and experimental manner. We can only avoid the extremes of harshness on the one hand and of weak indulgence on the other: but the man does not live who can feel sure of always steering a straight and steady middle course between these extremes. Some things we can accomplish. We can, and we ought to exercise more care than most of us do to see that free admission to hospitals is not given except to the really poor; and on the other hand, we ought to encourage and urge those who can pay, if only a little, to contribute to institutions by which they and their friends may some day be relieved.

Few Voluntary Supporters.

That leads me to notice what I have often mentioned before, the comparatively small number of people by whom medical charities and, indeed, all charities are supported. You will find a few who give even above their means, and many who give more or less liberally; but you will also find in every town that I know anything of that a very large proportion of the well-to-do altogether or almost wholly evade their duty in this respect. The tendency is strongest, I think, in very large towns, because there individual responsibility is neither so strongly felt nor so easily enforced by opinion. That is an evil in many ways, and it tends to grow because nobody likes to think that he is doing more than his duty only in order that somebody else may do less. And so there grows up a disposition to throw fresh charges on taxation because people say in that way nobody can escape, we are all made to pay alike, and that is fairest. So it is, but there are many incidental disadvantages, one being the soreness which follows on heavy taxation for whatever purpose imposed; another, perhaps more serious, that compulsory gifts produce no return of gratitude or good feeling on the part of those who receive, and involve no exercise of generosity on the part of those who give. The material results may be the same, but the moral results are widely different.

